# Anterior Mandibular Displacement in Growing Rats Enhances Growth—A 3D Analysis

**DOI:** 10.3390/bioengineering12090982

**Published:** 2025-09-16

**Authors:** Efstratios Ferdianakis, Ioannis Lyros, Demetrios Halazonetis, Georgios Kanavakis, Paula Perlea, Zafeiroula Yfanti, Konstantina-Eleni Alexiou, Dafni Doukaki, Apostolos I. Tsolakis

**Affiliations:** 1 Department of Orthodontics, School of Dentistry, National and Kapodistrian University of Athens, 11527 Athens, Greece; yiannislyros@hotmail.com (I.L.); dhal@dhal.com (D.H.); gkanavak@dent.uoa.gr (G.K.); atsolakis@dent.uoa.gr (A.I.T.); 2 Department of Endodontics, Carol Davila University of Medicine and Pharmacy, 050474 Bucharest, Romania; paula.perlea@gmail.com; 3 Department of Oral Diagnosis & Radiology, School of Dentistry, National and Kapodistrian University of Athens, 10679 Athens, Greece; zafeiroula86@gmail.com (Z.Y.); kalexiou@dent.uoa.gr (K.-E.A.); 4 Department of Orthodontics, Slovak Medical University, 83303 Bratislava, Slovakia; daphne.doukakis@gmail.com; 5 Department of Orthodontics, Case Western Reserve University, Cleveland, OH 44106, USA

**Keywords:** rats, anterior mandibular displacement, skeletal measurements

## Abstract

One of the most common malocclusions encountered in everyday practice by orthodontists is skeletal Class II malocclusion, namely a protrusion of the maxilla, a retrusion of the mandible or a combination of both. To correct it, many clinicians use functional devices that guide the mandible into a more forward position. This stimulates bone growth, correcting the skeletal discrepancy. Controversy exists as to whether these appliances accelerate the growth rate, helping the mandible reach its final size earlier, or whether the growth of the mandible is observed as a positive response to the stimuli. This study examined whether the protrusion of the mandible in rats accelerates the growth rate or increases the overall growth of the mandible in the long run. Relapse was also assessed by removing the appliance prior to the end of the experiment. Seventy-two four-week-old Wistar rats were used. The treatment group, which consisted of 36 rats, had a device fitted on their upper incisors that led to a protrusion of their mandible. The device, a bite-jumping appliance, consisted of an iron-cast inclined plane and was fitted for 24 h a day, inducing a 3.5 mm anterior protrusion and 3 mm inferior displacement of the mandible. The control group consisted of 36 rats that were fed the same soft diet as the treatment group. Both groups were divided into three subgroups. The first was sacrificed 30 days after the onset of the experiment, the second at 60 days, and the last subgroup had the appliance removed for 30 days and was sacrificed 90 days after the onset of the experiment. At the beginning of the experiment, as well as at each time interval prior to the sacrifice of the animals, the appliances were removed, and cone beam-computed tomography was performed on every animal. Linear measurements were made on each 3D scan, measuring the growth of the mandible. Measurements of mandibular growth were higher compared to the control group. For instance, Gonion-Menton was 1.18 mm higher on month 2 compared to month 1 in the control group, whereas the same measurement marked a 1.82 mm difference in the experimental group. Condylion–Menton on the same intervals marked a 0.84 mm difference in the control, whereas a 1.35 mm difference was noted in the experimental group. Given the results, true mandibular growth is achieved using functional appliances for Class II malocclusion correction in rats.

## 1. Introduction

### 1.1. Clinical Problem and Importance

Skeletal Class II malocclusion is a common orthodontic problem arising from an anteroposterior maxillomandibular discrepancy [1,2,3]. When due to mandibular retrognathia, stimulating mandibular growth is a primary treatment goal. Early orthopedic interventions, seek to guide skeletal growth during development.

### 1.2. Mechanistic Backround and Current Interventions

Functional orthodontic appliances have been shown to achieve this, even if only in the short term, by activating mandibular propulsive muscles [4,5], protruding the mandible, and thereby affecting condylar growth and modeling of the glenoid fossa [6,7,8,9,10,11,12,13]. The influence of functional alterations on the growth process, as well as on the morphology of the craniofacial complex, is of great interest to orthodontists. The relationship between function and skeletal form is one of the fundamental processes underlying craniofacial skeletal growth and development [14,15,16].

According to Frost (2004), bone form reflects cumulative mechanical loads [17]. Postnatally, strong musculature promotes the formation of stronger bones, joints, and tendons, while weak muscles result in weaker structures. In addition, loading applied to the tissues can change the shape of the cells. As a result, deformation of the intracellular content, including the cytoskeleton, is observed, and processes are activated that can even change the mechanisms of action of the genome [18]. The osteocyte network detects and responds to mechanical stimuli and thus plays an important role in triggering bone remodeling [19]. Despite these insights, a longstanding debate exists, regarding the extent to which functional activity affects the size, shape, and orientation of skeletal units [20]. Contributing factors to this controversy, include variability in methodology, measurement techniques, and follow-up, along with the ethical limitations of human trials [21,22,23,24]. Consequently, animal models remain essential for controlled investigation.

Current clinical practice in dentofacial orthopedics aims, by loading teeth and skeletal structures, not only to influence the dentoalveolar system but also to transmit therapeutic forces onto the adjacent bone and surrounding tissue. Various dentofacial orthopedic appliances are used in everyday clinical practice to enhance or retard maxillary and/or mandibular growth [25].

Even though several studies have demonstrated a positive response of the condyle to mandibular advancement, condylar growth as an effect of functional appliances has created controversy among researchers [9,26,27,28,29,30]. Some state that they simply accelerate the growth rate without contributing to an increase in the overall size of the mandible [5,31], while others demonstrate an actual size increase [32,33,34,35,36,37].

Given the fact that clinical trials on humans are restricted, as we mentioned above, rats are widely used as an experimental model, due existing similarities with humans regarding TMJ structures and condylar growth patterns, even though many morphological and functional differences exist. Rats can achieve free lateral movements of the mandible, and the rat’s mandibular resting position is approximately six millimeters posterior to the attritional position [38,39,40,41].

Remodeling of the condyle as a response to mandibular advancement in rats has been reported [42]. It has been shown that the use of fixed functional appliances advancing the mandible enhanced its growth [43]. Moreover, through this therapeutic approach, a significant increase in vascularization of condyle and mandibular bone growth was also reported in growing rats [42,44]. According to other studies, mandibular propulsive appliances increase cell proliferation, as well as the expression of IGF I, IGF II, and collagen-binding integrins in the condylar cartilage of growing rats [45,46].

Maintenance of the condylar cartilage is inextricably linked to mechanical forces. Optimal loading results in an increase in the anabolic response in chondrocytes [47]. In contrast, intense loading inhibits cell proliferation [48]. Neovascularization and osteogenesis induced by mechanical strain (produced by mandibular advancement) led to adaptive condyle growth in adult rats [49].

It has been reported that fibroblast growth factor 8 (FGF8) regulates new bone formation (mesenchymal and chondrocyte proliferation) in the condyles and glenoid fossae caused by mandibular advancement, resulting in condylar endochondral ossification and intramembranous ossification of the glenoid fossa [8,11,13,50]. Increased levels of Sox9 transcription factor and type II collagen expression have been reported during forward mandibular positioning, stating true enhancement of condylar growth. Sox9 regulates the differentiation of mesenchymal cells into chondrocytes, and type II collagen forms the framework of the new cartilage [9,11,51].

### 1.3. Research Gaps

Most animal studies assess only short-term effects of functional appliances, leaving long-term outcomes and post treatment relapse largely unexplored [9,10,27]. This study aimed to examine whether the protrusion of the mandible in rats accelerates the “preprogrammed” growth or increases the overall growth of the mandible in the long run. We also aimed to examine possible relapse following a period of appliance removal. Another important factor is that of image distortion inherent in standard lateral cephalometric radiographs, commonly used up to now by similar studies [52,53,54]. We aimed to overcome this limitation by measuring cone beam computed tomography images, which offer minimal distortion and greater anatomical accuracy.

## 2. Materials and Methods

The experimental protocol was approved by the Veterinary Directorate (598758/4 October 2019) and was registered in agreement with the Greek National legislation and the European Council.

### 2.1. Experimental Design

Six subgroups consisting of twelve rats were calculated using standard statistical criteria (a = 0.05, b = 0.10), yielding a power of 90% for detecting a 0.5 mm difference for the primary outcome of the study, namely mandibular length (Condylion–Menton). Seventy-two four-week-old Wistar rats were used in this experiment. They were bred and raised for their first four weeks in the Hellenic Institute of Pasteur. After this period, they were transferred to the Laboratory for Experimental Surgery and Surgical Research “N.S. Christeas”, located at the National and Kapodistrian University of Athens’ School of Medicine. They remained there until the completion of the experiment. They were kept in a stable environment, with a 55% humidity index, 20° Celsius temperature, and circadian artificial lighting of 12 h daytime and 12 h nighttime.

The animals were randomly allocated into two groups, the treatment group (A) and the control group (B). Each group consisted of three subgroups, A1, A2, and A3 and B1, B2, and B3, respectively, of twelve animals each. A1 and B1 were sacrificed thirty days after the onset of the experiment, A2 and B2 on day sixty, and at that time, the appliances fitted to the A3 group were removed. On day 90, the remaining animals, which consisted of subgroups A3 and B3, were sacrificed.

**Figure 1:**
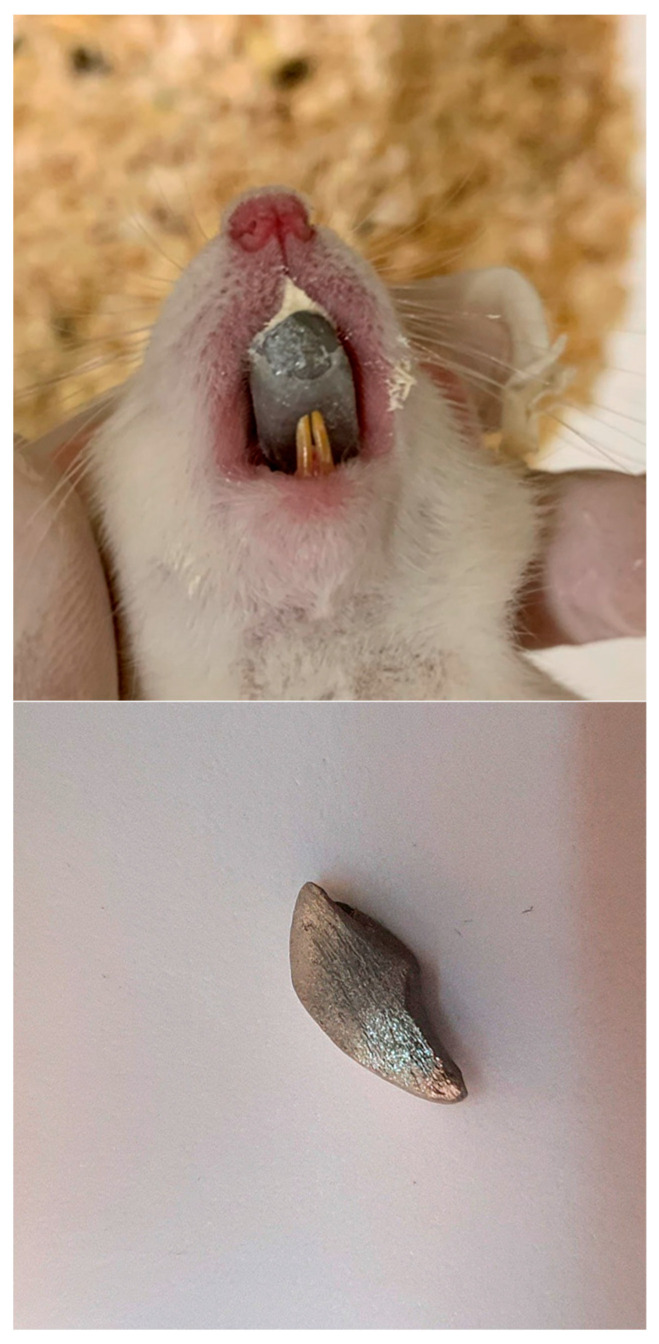
The appliance used in the experimental group.

The treatment group had a device fitted on their upper incisors that led to a protrusion of their mandible (Figure 1). The device was an iron-cast inclined plane. These bite-jumping appliances were fitted for 24 h a day and produced a 3.5 mm anterior and 3 mm inferior displacement of the mandible. They had identical inclination planes in order to provoke a fixed magnitude of downward and forward positioning of the mandible and, consequently, a downward and forward deviation of the condyle from the fossa. They were kept in place using zinc phosphate cement (Harvard Cements Harvad dental int., 15366 Hoppegarten, Germany).

**Figure 2:**
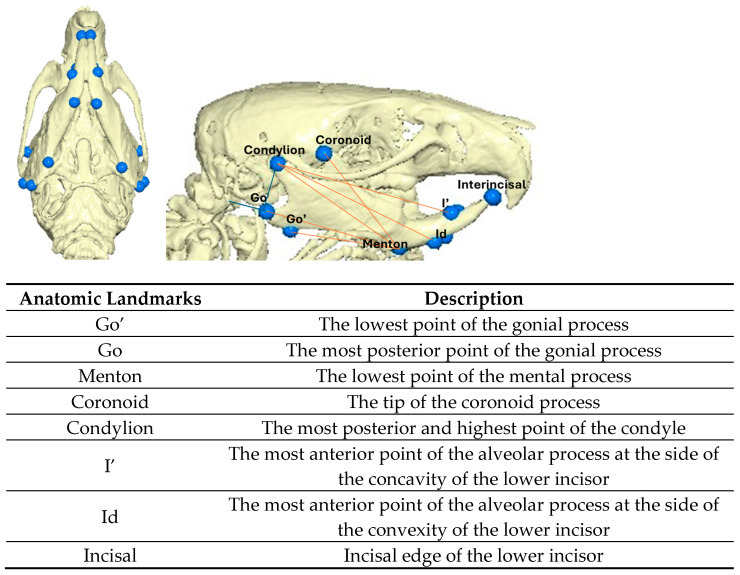
Anatomic landmarks and measurements made on CBCTs.

At each interval prior to the sacrifice of the animals, they were anesthetized (ketamine- xylazine combination 0.2 mL/kg intramuscularly), the appliances were removed, and cone beam computed tomography (CBCT) was performed on each animal. The CBCT was completed using the unit NewTom (VGi, Cefla SC, Imola, Italy) using the same field of view (8 × 8 cm, high-resolution, denture scan). Each scan was performed by an oral and dentomaxillofacial radiologist, who assessed the quality of the images taken. In case of artifact detection (i.e., small movements of the rats that had an effect on the final image), the scans were performed again so the final image would not have any artifacts that would interfere with proper measurements of the landmarks that were measured. The scans were saved as DICOM 3 datasets. Three-dimensional reconstruction and analysis were completed using the Viewbox software (Viewbox version 4.1.0.10, dHAL Software, Kifissia, Greece). The anatomic landmarks and linear measurements taken are depicted in Figure 2.

### 2.2. Statistical Analysis

To calculate the intra- and inter- observer errors, double measurements, 4 weeks apart were made by two independent and blinded observers. Lin’s concordance correlation coefficient, as well as the Bland and Altman limits of agreement, were used (Tables S1–S4) [55]. The distribution of the differences between the two measurements was assessed using histograms and Kolmogorov–Smirnov test for normality. The results show very good agreement between the measurements of both researchers.

Distribution characteristics, in terms of mean and standard deviation, for all parameters’ measurements in both control and treatment groups are presented in Table 1 and Table 2. The change in each parameter was calculated as the difference between the final and the initial measurements.

Differences in the change (final–initial) in each parameter were investigated using multiple linear regression models. Independent variables in the models were the duration of intervention (1, 2 or 3 months), the treatment group (intervention and control), and their interaction. The effect of initial weight was also considered as a confounding factor in the regression models. We applied pairwise post hoc tests to compare all categories, using Bonferroni correction for multiple comparisons.

Results are shown in tables and graphs as means, standard deviations, and 95% confidence intervals (95% CI).

All tests were two-sided, and the level of statistical significance was set at a = 5%.

Stata v13 was used for statistical analysis (StataCorp LP. College Station, TX, USA).

## 3. Results

Descriptive statistics for the control and treatment groups are depicted in Table 1 and Table 2, respectively. As observed in both tables, the initial measurements of all subgroups had no statistically significant differences between them, which implies that randomization was successful.

Statistical analysis of the measurements depicted in Table 3 shows an increased growth of the mandible in the treatment group compared to the control group. The differences in mandibular length measurements were larger in the treatment group. This difference was statistically significant with *p* < 0.001.

Statistically significant differences were observed in the change in almost every variable between the control and treatment groups at each interval, as well as in each subgroup at each time interval (Table 3 and Table 4).

**Figure 3:**
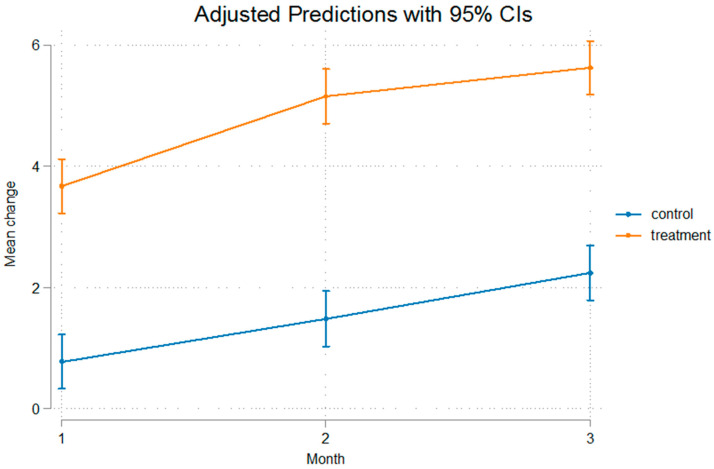
Recorded change between initial and final values and 95% confidence intervals of the “Go’ Menton” measurement. The treatment group showed a significantly greater increase compared to controls, with a steeper growth during appliance wear.

**Figure 4:**
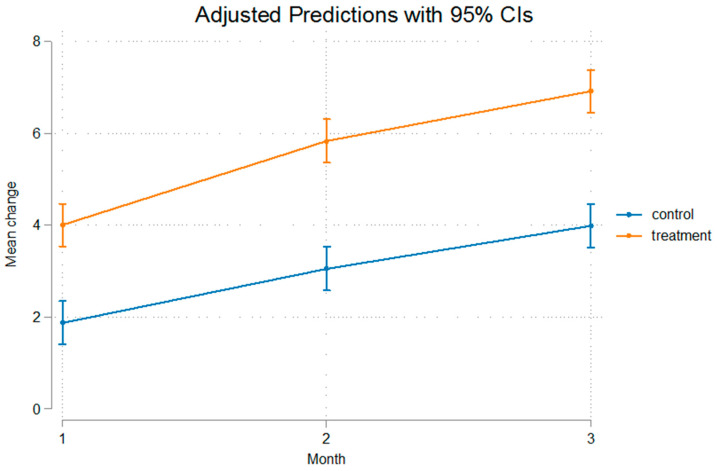
Recorded change between initial and final values and 95% confidence intervals of the “Go Menton” measurement. Growth was consistently higher in the treatment groups, indicating enhanced mandibular length increase.

The differences were statistically significant even after removal of the appliance that was used for a duration of 60 days, implying that after that no relapse was present, or at least, the relapse noted was not of such a measure that it could alter the outcome of mandibular advancement. The fact that Condylion-Menton at T3 shows a statistical significant difference between the experimental and control groups, measuring at 3.11 mm, and Condylion-I’ and Condylion-Id also show statistical significant differences of 2.84 mm and 2.36 mm, respectively (Table 3), imply that relapse was not present. As we can see in Figure 3 and Figure 4 the inclination from T2 to T3 on Go’-Menton and Go-Menton, respectively, is slightly less steep than that of T1–T2, which depicts a change in the growth pattern after appliance removal. The lines between control and treatment groups do not cross though, and the differences still remain statistically significant.

**Figure 5:**
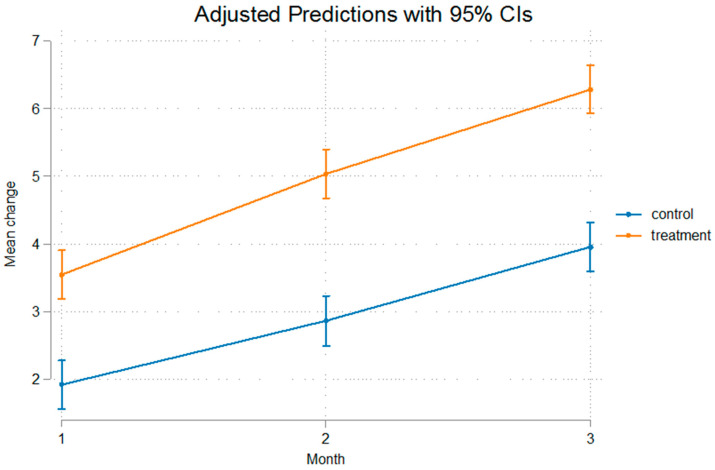
Recorded change between initial and final values and 95% confidence intervals of the “Coronoid Menton” measurement. The treatment group demonstrated a markedly greater increase, suggesting appliance-related stimulation of the coronoid process and mandible.

**Figure 6:**
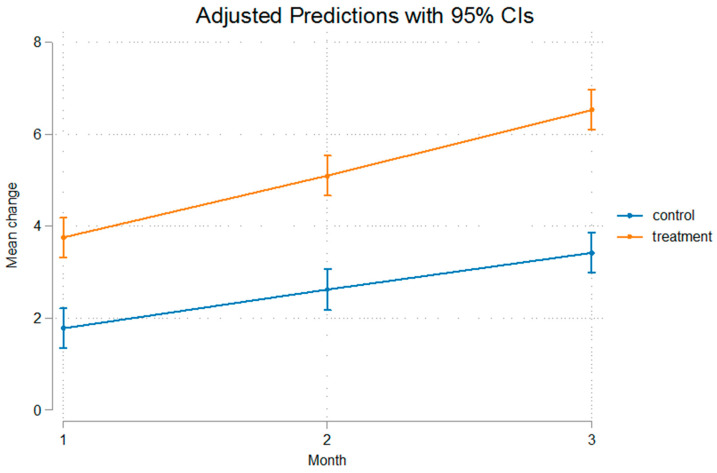
Recorded change between initial and final values and 95% confidence intervals of the “Condylion Menton” measurement. Growth acceleration was observed in the treatment group, indicating that the condyle contributes substantially to mandibular elongation.

**Figure 7:**
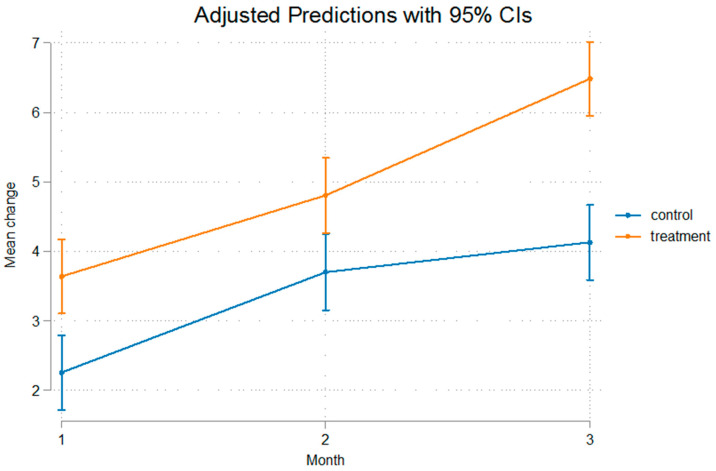
Recorded change between initial and final values and 95% confidence intervals of the “Condylion Id” measurement. Differences between treatment and control groups are more pronounced over time, implying the substantial role of the condyle in mandibular growth on the experimental group.

**Figure 8:**
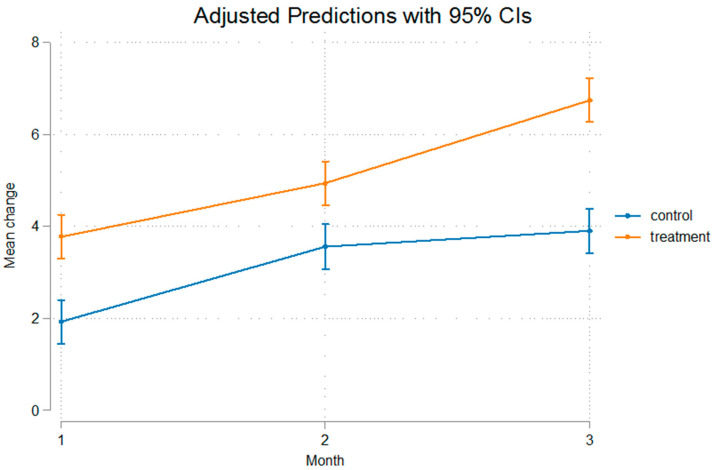
Recorded change between initial and final values and 95% confidence intervals of the “Condylion I’” measurement. Increase in growth after appliance removal suggests prolonged condylar activity.

The growth rate of the mandible was higher in the treatment group compared to the control group. This is illustrated in the figures below. In Figure 3 Gonion’-Menton not only exhibited a greater difference in the treatment group, but during appliance wear the rate of growth was also higher. Gonion-Menton and Coronoid-Menton show similar growth patterns in Figure 4 and Figure 5, respectively. Condylion-Menton, Condylion-Id and Condylion-I’ in Figure 6, Figure 7 and Figure 8 also showed an enhanced growth rate in the treatment group. Notably, the accelerated growth in these parameters continued even after the removal of the appliance. This sustained post-treatment growth implies that the condyle might be responsible for this ongoing mandibular development and remains active even after treatment at least for the 30-day period observed after appliance removal.

Gonion’-Menton and Gonion-Menton showed statistically significant differences between the control and the treatment groups at every time interval when measurements were taken and also posed similarities in their growth rate patterns, as depicted in Figure 7 and Figure 8, respectively.

## 4. Discussion

### 4.1. Comparison with Existing Literature

Mechanical stress is applied to the condyle and the mandible during the various growth stages, both through normal masticatory function and external interventions. Food consistency significantly influences mandibular development, with masticatory function changes capable of inducing endochondral ossification and bone apposition, or conversely, bone resorption. Functional appliances used in orthodontics for decades aim to alter the mandibular function and the strain imposed on it, leading its growth towards a certain path [56,57,58]. Some studies state that protrusive functional appliances increase the growth rate of the mandible and do not contribute at all to the actual increase in its final size [5]. Other studies support that growth is factual, as a positive response to the stimuli [32,33,34,35,36,37]. The response of the condyle to stimuli is depicted in the results of many studies changing the consistency of the diet in rats [59]. The current study shows that not only is mandibular growth achieved, but the growth rate of the mandible is also increased in the treatment group.

Shen et al. (2005) reported that the body of the mandible grows longer by periosteal apposition on its posterior surface, and the ramus grows taller mainly by endochondral replacement at the condyle [21]. Tagliaro et al. (2006) and Otwad et al. (2011) confirmed that condylar cartilage can be mechanically stimulated as well as by loading-induced growth factors [8,60]. Wang (2018) hypothesized synergistic effects between growth hormone therapy and functional appliance treatment demonstrating increased mandibular growth in adolescent rats [30]. Xiong et al. produced a mandibular protrusion with a bite-jumping appliance and found that the length of the condylar process increased significantly over a 30-day experimental period [49]. The present findings align with these studies, supporting the idea that functional appliances can produce true mandibular elongation, although the condyle itself was not directly assessed.

### 4.2. Mechanisms of Mandibular Growth

Hoyte and Enlow proposed that mandibular growth is mediated by neuromuscular adaptations in the stomatognathic system, leading to remodeling at muscle attachment sites [61,62]. Extirpation experiments have documented that certain morphologic elements of the mandible are specifically associated with muscles and directly diminish in size if these muscles are removed or are not functioning [63,64].

Anterior mandibular displacement has been shown to increase length through endochondral ossification at the condyle, and remodeling of the ramus, with bone resorption anteriorly, and apposition posteriorly [4,40,65,66]. Figure 3, Figure 4, Figure 5, Figure 6, Figure 7 and Figure 8 are in agreement with this statement, depicting a larger increase in mandibular length in the treatment group compared to the control group.

Controversy exists as to whether true mandibular growth happens due to the condyle acting as a growth site or due to remodeling of the fossae. Many state that changes in the mandible are due to both of the above. On the other hand, some attribute changes solely to dentoalveolar adaptations [67]. While previous studies have reported increased mandibular size due to hyperpropulsion, the contribution of the hypertrophy layer of the condylar cartilage specifically versus changes in the surrounding functional matrix remains vague. According to Killiany (1984), condylar loading results in decreased vertical condylar growth, while unloading results in an increase in vertical growth of the condyle [68]. Yozwiak (1979) and Tsolakis (1981) also demonstrated that it is possible to retard mandibular growth through loading [69,70].

### 4.3. Methodological Considerations

Experimental studies on mandibular advancement have yielded variable results, likely due to heterogeneity in appliance design and methodological factors such as the angle mandibular opening, displacement mode (continuous or intermittent), and the extent of anterior displacement [38,39,71,72]. To address these limitations, Tsolakis and Spyropoulos (1997) [38] introduced a custom-designed device that enabled controlled, stable and reproducible anterior displacement of the mandible in rats. This system applied a 25 g force for 12 h/day, over a 30-day period, simulating forced protrusion rather than functional repositioning. Although linear measurements revealed an increased mandibular length in the experimental group, the contribution of condylar cartilage remained uncertain [38]. The present supports the notion that functional appliance therapy can induce true mandibular growth, in agreement with previous findings, warranting further histological evaluation of the condylar cartilage. The statistically significant differences in measurements such as Gonion-Menton and Condylion-Menton observed in the current study reflect not only the elongation of the mandible but also the underlying biological adaptations, linked to endochondral ossification in the condylar cartilage and bone apposition on the posterior ramal part. These growth patterns are consistent with the time course of mechanically induced ossification reported in histological studies.

Rat occlusion differs markedly to that of animals such as monkeys or other primates. An approximately 10 mm edentulous space exists between molars and incisors, making interdigitating occlusion not feasible. Rats exhibit free rotational and lateral movement of the jaw, limiting the efficacy of conventionally inclined plans for anterior positioning. The resting position of the mandible is 6 mm posterior to the attrition position [38,72]. To compensate, we utilized an inclined plane much larger than the ones previously used in other studies. Because of difficulties in eating and drinking water, all experimental animals were fed a soft diet, and water was placed in bowls instead of the standard water bottles typically used in the cages of experimental animals.

Radiographic methodology also affects measurement accuracy. Many studies rely on lateral cephalometric radiographs, which are prone to distortion [73]. In the present study, three-dimensional computed tomography was used to obtain distortion-free measurements. According to a study comparing CBCTs, lateral cephalograms and direct measurements on dry skulls, CBCT is considered to have the same accuracy as direct craniometric measurements, while cephalometric measurements have many differences compared to direct craniometric measurements [74]. Studies directly comparing 2D and 3D imaging measurements on rat skulls also concluded that linear measurements are more reliable on 3D models [75].

### 4.4. Study Limitations

Future research could employ a device similar to the one that Tsolakis et al. use in their research [38], allowing for intermittent wear to assess compliance effects, thereby better simulating clinical orthodontic practice.

The age of the experimental animals used in these kinds of studies is of maximum importance. That is the reason rats used in the current study were all utilized immediately post-weaning aiming to capture maximal growth potential. Despite that, one study reported the formation of new bone even in adult rats [76].

While the present findings support others stating that true mandibular growth is achieved through functional appliance therapy [30,77], translation to humans must be made cautiously due to interspecies differences, and factors such as patient compliance. While the present study was limited to a growing rat model, future research directions could indeed strengthen the translational value. Specifically, longer follow-up periods would be important to assess the stability and persistence of the observed growth changes beyond the active treatment phase. Studies in higher-order animal models, such as primates, may also provide insights that are more directly comparable to human craniofacial development. Ultimately, well-designed clinical trials in growing patients will be necessary to confirm the biological mechanisms and treatment effects suggested by our findings. Moreover, incorporating objective methods for monitoring appliance wear and compliance in human studies would further enhance the reliability of outcomes.

## 5. Conclusions

Anterior mandibular displacement in growing rats resulted in significant and sustained increase in mandibular length, particularly in Gonion-Menton and Condylion-Menton measurements. These outcomes are consistent with endochondral ossification at the condylar cartilage and posterior ramal apposition induced by functional loading. The persistence of differences after appliance removal indicates that adaptive skeletal growth continues beyond the active treatment period.

## Figures and Tables

**Table 1:** Mean and standard deviation (SD) values for the initial and final measurements, as well as their difference for control group (B) subgroups.

Control Group	Mean (SD)	Mean (SD)	Mean (SD)	*p*-Value *
Subgroup	B1	B2	B3	
**Initial (mm)**				
**Weight (gr)**	109.42 (12.23)	105.42 (22.12)	121.67 (14.69)	0.062
Go’ Menton	12.80 (0.56)	13.04 (0.85)	12.95 (0.67)	0.698
Go Menton	16.51 (0.59)	16.67 (0.73)	16.75 (0.65)	0.668
Coronoid–Menton	16.06 (0.56)	16.07 (0.65)	16.18 (0.55)	0.850
Condylion/Go-Menton	8.02 (0.42)	8.20 (0.39)	8.43 (0.51)	0.090
Condylion–Menton	18.27 (0.68)	18.47 (0.71)	18.61 (0.62)	0.480
Condylion-Id	20.46 (0.74)	20.36 (0.84)	20.92 (0.85)	0.213
Condylion-I’	21.13 (0.85)	21.04 (0.91)	21.55 (0.63)	0.277
**Final (mm)**				
**Weight (gr)**	282.25 (18.14)	362.27 (35.06)	430.17 (30.11)	**<0.001**
Go’ Menton	13.65 (0.47)	14.65 (0.45)	15.10 (0.74)	**<0.001**
Go Menton	18.44 (0.66)	19.82 (0.59)	20.67 (0.66)	**<0.001**
Coronoid–Menton	18.05 (0.52)	19.06 (0.67)	20.05 (0.53)	**<0.001**
Condylion/Go-Menton	9.25 (0.39)	10.20 (0.58)	10.36 (0.41)	**<0.001**
Condylion–Menton	20.13 (0.71)	21.21 (0.74)	21.95 (0.59)	**<0.001**
Condylion-Id	22.80 (0.72)	24.21 (0.72)	24.96 (0.83)	**<0.001**
Condylion-I’	23.16 (0.68)	24.78 (0.61)	25.33 (0.53)	**<0.001**
**Difference (mm)**				
Go’ Menton	0.85 (0.70)	1.62 (0.84)	2.15 (0.84)	**0.001**
Go Menton	1.93 (0.81)	3.15 (0.89)	3.93 (0.88)	**<0.001**
Coronoid–Menton	2.00 (0.68)	2.99 (0.59)	3.87 (0.77)	**<0.001**
Condylion/Go-Menton	1.23 (0.46)	2.00 (0.64)	1.93 (0.40)	**0.001**
Condylion–Menton	1.85 (0.80)	2.74 (0.71)	3.35 (0.76)	**<0.001**
Condylion-Id	2.34 (0.87)	3.85 (0.62)	4.04 (1.25)	**<0.001**
Condylion-I’	2.03 (1.03)	3.74 (0.66)	3.78 (0.86)	**<0.001**

* one-way ANOVA.

**Table 2:** Mean and standard deviation values for the initial and final measurements, as well as their difference for treatment group (A) subgroups.

Treatment Group	Mean (SD)	Mean (SD)	Mean (SD)	
Subgroup Initial Measurements (mm)	A1	A2	A3	*p*-Value *
**Weight (gr)**	119.25 (13.71)	121.75 (11.92)	113.92 (14.90)	0.363
Go’ Menton	11.04 (0.40)	11.06 (0.72)	11.36 (0.62)	0.341
Go Menton	15.16 (0.42)	15.20 (0.68)	15.29 (0.67)	0.876
Coronoid–Menton	15.05 (0.63)	15.05 (0.50)	15.11 (0.44)	0.945
Condylion/Go-Menton	8.55 (0.42)	8.55 (0.44)	8.36 (0.40)	0.442
Condylion–Menton	16.98 (0.53)	17.00 (0.53)	17.06 (0.57)	0.929
Condylion-Id	19.45 (0.70)	19.61 (0.56)	19.55 (0.78)	0.858
Condylion-I’	19.76 (0.63)	19.90 (0.49)	19.74 (0.62)	0.747
**Final measurements (mm)**				
**Weight (gr)**	182.25 (23.49)	245.42 (36.08)	315.92 (38.04)	**<0.001**
Go’ Menton	14.66 (0.54)	16.12 (0.64)	17.01 (0.90)	**<0.001**
Go Menton	19.13 (0.55)	20.97 (0.69)	22.22 (0.55)	**<0.001**
Coronoid–Menton	18.54 (0.48)	20.00 (0.39)	21.41 (0.49)	**<0.001**
Condylion/Go-Menton	9.32 (0.50)	10.06 (0.44)	10.70 (0.82)	**<0.001**
Condylion–Menton	20.69 (0.52)	22.02 (0.55)	23.61 (0.60)	**<0.001**
Condylion-Id	23.04 (0.41)	24.32 (0.67)	26.05 (0.87)	**<0.001**
Condylion-I’	23.46 (0.53)	24.72 (0.56)	26.50 (0.89)	**<0.001**
**Difference (mm)**				
Go’ Menton	3.62 (0.69)	5.07 (0.72)	5.65 (0.92)	**<0.001**
Go Menton	3.97 (0.69)	5.77 (0.62)	6.93 (0.94)	**<0.001**
Coronoid–Menton	3.49 (0.61)	4.95 (0.54)	6.30 (0.66)	**<0.001**
Condylion/Go-Menton	0.77 (0.38)	1.50 (0.45)	2.34 (0.85)	**<0.001**
Condylion–Menton	3.70 (0.73)	5.03 (0.63)	6.55 (0.89)	**<0.001**
Condylion-Id	3.58 (0.72)	4.71 (0.89)	6.50 (1.12)	**<0.001**
Condylion-I’	3.70 (0.73)	4.82 (0.80)	6.76 (1.01)	**<0.001**

* one-way ANOVA.

**Table 3:** Mean differences and 95% confidence intervals (with Bonferroni correction for multiple comparisons) derived from multiple linear regression models between treatment group (A) and control groups (B), at each interval of the experiment.

	Mean Difference in mm	95% CI *	*p*-Value *
**Go’ Menton**			
T 1	2.90	(2.12, 3.69)	**<0.001**
T 2	3.67	(2.86, 4.48)	**<0.001**
T 3	3.39	(2.61, 4.17)	**<0.001**
**Go Menton**			
T 1	2.13	(1.31, 2.95)	**<0.001**
T 2	2.78	(1.93, 3.62)	**<0.001**
T 3	2.93	(2.12, 3.75)	**<0.001**
**Coronoid–Menton**			
T 1	1.63	(0.99, 2.26)	**<0.001**
T 2	2.17	(1.52, 2.82)	**<0.001**
T 3	2.33	(1.70, 2.96)	**<0.001**
**Condylion/Go-Menton**			
T 1	−0.44	(−1.01, 0.13)	0.186
T 2	−0.47	(−1.05, 0.12)	0.166
T 3	0.39	(−0.17, 0.96)	0.275
**Condylion–Menton**			
T 1	1.97	(1.21, 2.73)	**<0.001**
T 2	2.48	(1.69, 3.26)	**<0.001**
T 3	3.11	(2.36, 3.87)	**<0.001**
**Condylion-Id**			
T 1	1.38	(0.44, 2.32)	**0.002**
T 2	1.10	(0.13, 2.07)	**0.020**
T 3	2.36	(1.42, 3.29)	**<0.001**
**Condylion-I’**			
T 1	1.85	(1.01, 2.69)	**<0.001**
T 2	1.37	(0.51, 2.24)	**0.001**
T 3	2.84	(2.01, 3.67)	**<0.001**

* Bonferroni corrected.

**Table 4:** Mean differences and 95% confidence intervals (with Bonferroni correction for multiple comparisons) derived from multiple linear regression models between time intervals in the treatment group (A) and control group (B).

	Mean Difference (mm)	95% CI *	*p*-Value *
**Go’ Menton**			
T 2 vs. 1, Β	0.71	(−0.10, 1.52)	0.111
T 3 vs. 1, Β	1.47	(0.64, 2.30)	**<0.001**
T 3 vs. 2, Β	0.76	(−0.09, 1.60)	0.097
T 2 vs. 1, A	1.48	(0.67, 2.29)	**<0.001**
T 3 vs. 1, A	1.95	(1.14, 2.76)	**<0.001**
T 3 vs. 2, A	0.47	(−0.34, 1.29)	0.565
**Go Menton**			
T 2 vs. 1, Β	1.18	(0.33, 2.02)	**0.003**
T 3 vs. 1, Β	2.11	(1.24, 2.98)	**<0.001**
T 3 vs. 2, Β	0.93	(0.05, 1.82)	**0.034**
T 2 vs. 1, A	1.82	(0.98, 2.67)	**<0.001**
T 3 vs. 1, A	2.91	(2.07, 3.76)	**<0.001**
T 3 vs. 2, A	1.09	(0.24, 1.94)	**0.007**
**Coronoid–Menton**			
T 2 vs. 1, Β	0.94	(0.29, 1.59)	**0.002**
T 3 vs. 1, Β	2.03	(1.37, 2.70)	**<0.001**
T 3 vs. 2, Β	1.09	(0.41, 1.77)	**<0.001**
T 2 vs. 1, A	1.49	(0.84, 2.14)	**<0.001**
T 3 vs. 1, A	2.74	(2.09, 3.39)	**<0.001**
T 3 vs. 2, A	1.25	(0.59, 1.90)	**<0.001**
**Condylion/Go-Menton**			
T 2 vs. 1, Β	0.76	(0.18, 1.35)	**0.006**
T 3 vs. 1, Β	0.73	(0.12, 1.33)	**0.011**
T 3 vs. 2, Β	−0.04	(−0.65, 0.58)	>0.999
T 2 vs. 1, A	0.74	(0.15, 1.32)	**0.008**
T 3 vs. 1, A	1.56	(0.97, 2.15)	**<0.001**
T 3 vs. 2, A	0.82	(0.23, 1.42)	**0.003**
**Condylion–Menton**			
T 2 vs. 1, Β	0.84	(0.06, 1.62)	**0.030**
T 3 vs. 1, Β	1.64	(0.83, 2.44)	**<0.001**
T 3 vs. 2, Β	0.79	(−0.02, 1.61)	0.060
T 2 vs. 1, A	1.35	(0.57, 2.13)	**<0.001**
T 3 vs. 1, A	2.78	(2.00, 3.56)	**<0.001**
T 3 vs. 2, A	1.43	(0.64, 2.22)	**<0.001**
**Condylion-Id**			
T 2 vs. 1, Β	1.45	(0.48, 2.41)	**0.001**
T 3 vs. 1, Β	1.87	(0.88, 2.86)	**<0.001**
T 3 vs. 2, Β	0.43	(−0.59, 1.44)	>0.999
T 2 vs. 1, A	1.17	(0.20, 2.13)	**0.011**
T 3 vs. 1, A	1.87	(0.88, 2.86)	**<0.001**
T 3 vs. 2, A	1.68	(0.70, 2.65)	**<0.001**
**Condylion-I’**			
T 2 vs. 1, Β	1.64	(0.77, 2.50)	**<0.001**
T 3 vs. 1, Β	1.97	(1.09, 2.86)	**<0.001**
T 3 vs. 2, Β	0.34	(−0.57, 1.24)	>0.999
T 2 vs. 1, A	1.16	(0.30, 2.02)	**0.004**
T 3 vs. 1, A	2.96	(2.10, 3.83)	**<0.001**
T 3 vs. 2, A	1.80	(0.93, 2.67)	**<0.001**

* Bonferroni corrected.

## Data Availability

The data presented in this study are available on request from the corresponding author.

## Supplementary Materials

The following supporting information can be downloaded at: https://www.mdpi.com/article/10.3390/bioengineering12090982/s1. Table S1. Lin’s concordance correlation coefficient (CCC), mean difference, and Bland–Altman 95% limits of agreement (LOA) between the first and second measurements of the first examiner for the initial measurements of the control and the experimental groups. Table S2. Lin’s concordance correlation coefficient (CCC), mean difference, and Bland–Altman 95% limits of agreement (LOA) between the first and second measurements of the first examiner for the final measurements of the control and the experimental groups. Table S3. Lin’s concordance correlation coefficient (CCC), mean difference, and Bland–Altman 95% limits of agreement (LOA) between the first and second examiners for the initial measurements of the control and the experimental groups. Table S4. Lin’s concordance correlation coefficient (CCC), mean difference, and Bland–Altman 95% limits of agreement (LOA) between the first and second examiners for the final measurements of the control and the experimental groups.
