# Supplementary material for: Anterior Mandibular Displacement in Growing Rats Enhances Growth—A 3D Analysis
Source: Bioengineering (Basel). 2025 Sep 16;12(9):982. doi: 10.3390/bioengineering12090982 (PMC12466957; doi:10.3390/bioengineering12090982)
Supplement: Supplementary file 1 [file bioengineering-12-00982-s001.zip › bioengineering-3713167-supplementary.docx]

**Table S1.** Lin’s concordance correlation coefficient (CCC), mean difference, and Bland–Altman 95% limits of agreement (LOA) between the first and second measurements of the first examiner for the initial measurements of the control and the experimental groups.

|  | CCC |  | Mean diff. | 95% LOA(mm) |
| --- | --- | --- | --- | --- |
| Go’-Menton right (mm) | 0.945 |  | 0.108 | (-0.570, 0.787) |
| Go-Menton right (mm) | 0.948 |  | 0.035 | (-0.566, 0.635) |
| Go’-Menton left (mm) | 0.948 |  | 0.154 | (-0.575, 0.883) |
| Go-Menton left (mm) | 0.959 |  | 0.053 | (-0.528, 0.634) |
| Coronoid-Menton right (mm) | 0.973 |  | 0.010 | (-0.336, 0.355) |
| Coronoid–Menton left (mm) | 0.977 |  | 0.019 | (-0.319, 0.358) |
| Condylion/Go-Menton right (mm) | 0.861 |  | 0.056 | (-0.442, 0.553) |
| Condylion/Go-Menton left (mm) | 0.902 |  | 0.096 | (-0.278, 0.470) |
| Condylion–Menton right (mm) | 0.972 |  | 0.015 | (-0.426, 0.456) |
| Condylion–Menton left (mm) | 0.975 |  | 0.031 | (-0.399, 0.460) |
| Condylion-Ιd right (mm) | 0.979 |  | -0.046 | (-0.399, 0.307) |
| Condylion-Id left (mm) | 0.973 |  | -0.029 | (-0.443, 0.385) |
| Condylion-I’ right (mm) | 0.987 |  | -0.003 | (-0.328, 0.323) |
| Condylion-I’ left (mm) | 0.986 |  | -0.006 | (-0.344, 0.333) |
| Average left–right |  |  |  |  |
| Go’-Menton (mm) | 0.956 |  | 0.131 | (-0.475, 0.737) |
| Go-Menton (mm) | 0.959 |  | 0.044 | (-0.506, 0.593) |
| Coronoid–Menton (mm) | 0.979 |  | 0.015 | (-0.290, 0.319) |
| Condylion/Go-Menton (mm) | 0.906 |  | 0.076 | (-0.281, 0.433) |
| Condylion–Menton (mm) | 0.978 |  | 0.023 | (-0.369, 0.415) |
| Condylion-Id (mm) | 0.984 |  | -0.037 | (-0.344, 0.269) |
| Condylion-I’ (mm) | 0.989 |  | -0.004 | (-0.297, 0.289) |

**Table S2.** Lin’s concordance correlation coefficient (CCC), mean difference, and Bland–Altman 95% limits of agreement (LOA) between the first and second measurements of the first examiner for the final measurements of the control and the experimental groups.

|  | CCC |  | Mean diff. | 95% LOA(mm) |
| --- | --- | --- | --- | --- |
| Go-Menton right (mm) | 0.917 |  | 0.050 | (-0.927, 10.027) |
| Go-Menton right (mm) | 0.953 |  | 0.099 | (-0.665, 0.862) |
| Go’-Menton left (mm) | 0.938 |  | 0.165 | (-0.744, 10.075) |
| Go-Menton left (mm) | 0.950 |  | 0.096 | (-0.770, 0.962) |
| Coronoid–Menton right (mm) | 0.974 |  | 0.054 | (-0.459, 0.567) |
| Coronoid–Menton left (mm) | 0.960 |  | 0.075 | (-0.613, 0.763) |
| Condylion/Go-Menton right (mm) | 0.949 |  | -0.024 | (-0.554, 0.506) |
| Condylion/Go-Menton left (mm) | 0.941 |  | 0.026 | (-0.466, 0.519) |
| Condylion–Menton right (mm) | 0.967 |  | 0.072 | (-0.526, 0.670) |
| Condylion–Menton left (mm) | 0.941 |  | 0.083 | (-0.776, 0.942) |
| Condylion-Id right (mm) | 0.965 |  | 0.083 | (-0.600, 0.767) |
| Condylion-Id left (mm) | 0.952 |  | 0.069 | (-0.734, 0.873) |
| Condylion-I’ right (mm) | 0.964 |  | 0.063 | (-0.629, 0.754) |
| Condylion-I’ left (mm) | 0.929 |  | 0.087 | (-0.879, 10.054) |
| Average left–right |  |  |  |  |
| Go’-Menton (mm) | 0.942 |  | 0.108 | (-0.701, 0.917) |
| Go-Menton (mm) | 0.956 |  | 0.097 | (-0.668, 0.863) |
| Coronoid–Menton (mm) | 0.971 |  | 0.065 | (-0.492, 0.621) |
| Condylion/Go-Menton (mm) | 0.955 |  | 0.001 | (-0.446, 0.449) |
| Condylion–Menton (mm) | 0.960 |  | 0.078 | (-0.598, 0.754) |
| Condylion-Id (mm) | 0.963 |  | 0.076 | (-0.612, 0.765) |
| Condylion-I’ (mm) | 0.952 |  | 0.075 | (-0.697, 0.847) |

**Table S3.** Lin’s concordance correlation coefficient (CCC), mean difference, and Bland–Altman 95% limits of agreement (LOA) between the first and second examiners for the initial measurements of the control and the experimental groups.

|  | CCC |  | Mean diff. | 95% LOA(mm) |
| --- | --- | --- | --- | --- |
| Go’-Menton right (mm) | 0.892 |  | 0.144 | (-0.832, 10.121) |
| Go-Menton right (mm) | 0.919 |  | -0.028 | (-0.776, 0.720) |
| Go’-Menton left (mm) | 0.911 |  | 0.215 | (-0.745, 10.176) |
| Go-Menton left (mm) | 0.939 |  | 0.007 | (-0.709, 0.723) |
| Coronoid–Menton right (mm) | 0.956 |  | 0.000 | (-0.447, 0.447) |
| Coronoid–Menton left (mm) | 0.953 |  | -0.010 | (-0.490, 0.471) |
| Condylion/Go-Menton right (mm) | 0.838 |  | 0.065 | (-0.455, 0.586) |
| Condylion/Go-Menton left (mm) | 0.833 |  | 0.108 | (-0.383, 0.599) |
| Condylion–Menton right (mm) | 0.954 |  | -0.026 | (-0.591, 0.539) |
| Condylion–Menton left (mm) | 0.959 |  | -0.011 | (-0.563, 0.541) |
| Condylion-Id right (mm) | 0.956 |  | -0.031 | (-0.552, 0.491) |
| Condylion-Id left (mm) | 0.952 |  | -0.056 | (-0.589, 0.478) |
| Condylion-I’ right (mm) | 0.982 |  | 0.011 | (-0.373, 0.396) |
| Condylion-I’ left (mm) | 0.983 |  | -0.035 | (-0.394, 0.324) |
| Average left–right |  |  |  |  |
| Go’-Menton (mm) | 0.916 |  | 0.180 | (-0.688, 10.048) |
| Go-Menton (mm) | 0.936 |  | -0.010 | (-0.703, 0.682) |
| Coronoid–Menton (mm) | 0.958 |  | -0.005 | (-0.439, 0.429) |
| Condylion/Go-Menton (mm) | 0.859 |  | 0.087 | (-0.340, 0.513) |
| Condylion–Menton (mm) | 0.962 |  | -0.019 | (-0.539, 0.501) |
| Condylion-Id (mm) | 0.963 |  | -0.043 | (-0.505, 0.419) |
| Condylion-I’ (mm) | 0.989 |  | -0.012 | (-0.309, 0.285) |

**Table S4.** Lin’s concordance correlation coefficient (CCC), mean difference, and Bland–Altman 95% limits of agreement (LOA) between the first and second examiners for the final measurements of the control and the experimental groups.

|  | CCC |  | Mean diff. | 95% LOA(mm) |
| --- | --- | --- | --- | --- |
| Go’-Menton right (mm) | 0.905 |  | 0.038 | (-0.996, 10.071) |
| Go-Menton right (mm) | 0.947 |  | 0.082 | (-0.735, 0.899) |
| Go’-Menton left (mm) | 0.923 |  | 0.168 | (-0.827, 10.163) |
| Go-Menton left (mm) | 0.941 |  | 0.056 | (-0.882, 0.993) |
| Coronoid–Menton right (mm) | 0.970 |  | 0.075 | (-0.470, 0.620) |
| Coronoid–Menton left (mm) | 0.954 |  | 0.071 | (-0.655, 0.797) |
| Condylion/Go-Menton right (mm) | 0.949 |  | -0.011 | (-0.542, 0.520) |
| Condylion/Go-Menton left | 0.921 |  | 0.054 | (-0.510, 0.618) |
| Condylion–Menton right (mm) | 0.958 |  | 0.083 | (-0.596, 0.763) |
| Condylion–Menton left (mm) | 0.940 |  | 0.096 | (-0.754, 0.946) |
| Condylion-Id right (mm) | 0.955 |  | 0.103 | (-0.665, 0.871) |
| Condylion-Id left (mm) | 0.948 |  | 0.040 | (-0.799, 0.88) |
| Condylion-I’ right (mm) | 0.960 |  | 0.078 | (-0.651, 0.807) |
| Condylion-I’ left (mm) | 0.926 |  | 0.093 | (-0.900, 10.086) |
| Average left–right |  |  |  |  |
| Go’-Menton (mm) | 0.932 |  | 0.103 | (-0.760, 0.965) |
| Go-Menton (mm) | 0.949 |  | 0.069 | (-0.762, 0.900) |
| Coronoid–Menton (mm) | 0.966 |  | 0.073 | (-0.524, 0.669) |
| Condylion/Go-Menton (mm) | 0.948 |  | 0.022 | (-0.457, 0.500) |
| Condylion–Menton (mm) | 0.955 |  | 0.090 | (-0.623, 0.803) |
| Condylion-Id (mm) | 0.957 |  | 0.072 | (-0.672, 0.815) |
| Condylion-I’ (mm) | 0.952 |  | 0.085 | (-0.688, 0.858) |
